# NANOG Proximity Proteomics Maps Neighborhood Hubs Linked to Mesenchymal Stem Cell Stemness and Chromatin Control

**DOI:** 10.3390/biom16040531

**Published:** 2026-04-02

**Authors:** Kyoung-Jae Choi, Michail Tyryshkin, Harathi Jonnagaddala, Allan Chris M. Ferreon, Marian Kalocsay, Josephine C. Ferreon

**Affiliations:** 1 Department of Biochemistry and Molecular Pharmacology, Baylor College of Medicine, Houston, TX 77030, USA; 2 Department of Experimental Radiation Oncology, The University of Texas MD Anderson Cancer Center, Houston, TX 77030, USA

**Keywords:** NANOG, mesenchymal stem/stromal cells, rejuvenation, aging, APEX, proximity proteomics, RNA processing, chromatin remodeling, DNA replication, transcription elongation, mechanotransduction

## Abstract

NANOG overexpression has been reported to reverse aging-associated decline in mesenchymal stem/stromal cell (MSC) function, but the molecular machinery engaged by NANOG in MSCs remains incompletely defined. Here, we applied APEX proximity labeling coupled with quantitative mass spectrometry to define the NANOG proximity interactome (proxeome) in human MSCs. Of 1040 quantified proteins, 828 were significantly enriched in the APEX-NANOG (H_2_O_2_ labeling) samples, consistent with a broad NANOG-centered neighborhood rather than a single stoichiometric complex. Enriched proteins encompass RNA-processing pathways (including splicing/RNP factors and selected m6A-related proteins), transcriptional coactivation and elongation control (Mediator and 7SK/P-TEFb regulators), chromatin repression/poising modules (Polycomb and HDAC/NuRD/CoREST/SIN3), ATP-dependent chromatin remodeling (BAF/SWI-SNF), three-dimensional genome organization and replication-coupled chromatin maintenance (CTCF/cohesin, CHAF1A, RIF1, UHRF1), and regulators of MSC identity and signal integration (Hippo/mechanotransduction and TGFβ-linked transcriptional circuits). Together, these data provide a spatial proteomic map of NANOG-associated nuclear neighborhoods in MSCs and a foundation for mechanistic hypotheses for how NANOG may stabilize stem-like programs.

## 1. Introduction

Mesenchymal stem/stromal cells (MSCs) represent a significant population of adult stem-like cells with substantial clinical interest due to their multipotent differentiation capacity and potent immunomodulatory properties [1,2]. However, MSCs display considerable heterogeneity at both the population and single-cell levels, exhibiting variability in surface marker expression, lineage preferences, and functional outcomes [1,2]. MSC fate and function are also highly sensitive to environmental influences, including mechanical cues, extracellular matrix characteristics, and signaling through pathways such as YAP/TEAD and TGFβ/SMAD [3]. These factors significantly impact MSC biology, particularly in the context of aging.

Aging, chronic inflammation, and prolonged culture expansion collectively drive MSCs toward senescence, reducing their clonogenicity, differentiation potential, and therapeutic efficacy [4]. Such functional declines have motivated research into genetic and pharmacological strategies aimed at rejuvenating MSC function. Notably, forced expression of the embryonic transcription factor NANOG has been shown to reverse age-associated functional deterioration in adult bone marrow-derived MSCs. Studies report restoration of proliferative capabilities and myogenic differentiation potential, consequently enhancing the functionality of engineered tissues [5,6]. These findings position NANOG as a promising candidate for stem cell rejuvenation. Nonetheless, the underlying molecular mechanisms through which NANOG achieves these regenerative effects within the distinct chromatin environment and signaling landscape of MSCs remain insufficiently understood.

NANOG was originally identified in mouse embryonic stem cells as a homeodomain transcription factor required for pluripotency and epiblast development, and remains a central node of pluripotency transcriptional networks [7,8,9,10]. In embryonic stem cells, NANOG participates in dense interaction neighborhoods with chromatin regulators and the transcription machinery, including the Nanog/Oct4-associated deacetylase (NODE) complex and SWI/SNF remodeling modules [11,12,13]. Despite this comprehensive characterization in pluripotent cells, the molecular mechanisms underlying NANOG’s rejuvenation potential in MSCs, particularly within mesenchymal chromatin and signaling contexts, remain less clearly defined. Moreover, most interaction studies have been conducted using mouse interactors, while human-related interactors have been less explored, highlighting important differences in the biophysical properties of NANOG across species that could influence its behavior [14,15,16].

Although MSCs are not pluripotent, multiple studies support the functional relevance of NANOG in MSC-like states. Endogenous NANOG expression is typically lower and more heterogeneous in MSC cultures than in ESCs, but it is detectable and linked to MSC self-renewal-associated transcriptional programs. For example, OCT4 and NANOG directly regulate DNMT1 in MSCs, coupling pluripotency-factor activity to epigenetic maintenance of an undifferentiated state [17]. In addition, Wnt/β-catenin accumulation is associated with increased NANOG expression and sustained MSC self-renewal [18]. These observations suggest that NANOG abundance in MSCs, whether endogenous or induced, can effectively activate transcriptional and chromatin regulatory mechanisms critical for maintaining stemness.

To further elucidate the proteins involved in NANOG-induced effects, we utilized APEX proximity labeling, a technique that captures local protein neighborhoods in living cells. APEX catalyzes proximity-dependent biotinylation over a short labeling pulse, enabling enrichment and identification of proteins within a restricted spatial radius of a bait protein, including transient and weak interactions [19]. In this context, we refer to the set of proteins labeled around NANOG as the NANOG proxeome (proximity interactome), emphasizing that the readout reflects spatial neighborhood rather than exclusively direct binding.

## 2. Materials and Methods

### 2.1. Construction of the pLVX-mApple-APEX-NANOG Expression Vector

A doxycycline-inducible lentiviral expression vector was generated using pLVX-Ubc-rtTA (Addgene plasmid #127288) as the cloning backbone. The NANOG coding sequence was amplified by PCR from pEP4-E02S-EN2L (Addgene plasmid #20922). In parallel, the mApple-APEX fragment was amplified from pIN10-mApple-APEX-MBNL1. The mApple-APEX and NANOG fragments were assembled sequentially and in-frame to generate an N-terminal fluorescent APEX fusion (mApple-APEX-NANOG), yielding the final transfer plasmid pLVX-mApple-APEX-NANOG. Correct insert orientation, fusion junctions, and the open reading frame were verified by DNA sequencing.

### 2.2. Lentivirus Production and Transduction of Mesenchymal Stem Cells

Lentiviral particles were produced by transient co-transfection of Lenti-X 293T cells (Takara Bio USA, San Jose, CA, USA) with the pLVX-mApple-APEX-NANOG transfer plasmid together with the packaging plasmids psPAX2 and pMD2.G (standard second-generation packaging system). Viral supernatants were collected, clarified by centrifugation, and filtered prior to concentration. Virus was concentrated 100-fold using 5× Lenti Concentration Solution (OriGene Technologies, Rockville, MD, USA, Cat. #TR30025) according to the manufacturer’s instructions.

Human mesenchymal stem cells (MSCs; ATCC SCRC-4000) were cultured in DMEM/F12 supplemented with 10% fetal bovine serum (FBS), 1× GlutaMAX, 1× antibiotic-antimycotic, and 0.2 mg/mL G418 to maintain hTERT expression. Cells were transduced with concentrated lentivirus at a multiplicity of infection (MOI) of 10 in the presence of 8 µg/mL polybrene. Following transduction, cells were selected with puromycin (2 µg/mL) to establish stable cell populations; for routine maintenance, puromycin was reduced to 1 µg/mL. Expression of the mApple-APEX-NANOG fusion protein was induced by treating cells with doxycycline (2 µg/mL) for 48 h and was verified by mApple fluorescence.

### 2.3. APEX-Mediated Proximity Biotinylation

APEX labeling was performed essentially as described for peroxidase-based proximity biotinylation [20], with minor modifications. Two experimental conditions were analyzed: (i) a −H_2_O_2_ control in which biotin-tyramide was added but H_2_O_2_ was omitted, and (ii) a +H_2_O_2_ labeling condition that received H_2_O_2_ for 45 s to initiate APEX-catalyzed biotinylation. Importantly, in both conditions, MSCs were doxycycline-induced to express mApple-APEX-NANOG; the −H_2_O_2_ control, therefore, captures non-specific streptavidin enrichment and any H_2_O_2_-independent background biotinylation under the same fusion-protein expression state. For each condition, three independent biological replicates were prepared, and each biological replicate consisted of material collected from three parallel 10 cm dishes.

For APEX labeling, MSCs were incubated in 4 mL of fresh culture medium containing 500 µM biotin-tyramide for 1 h at 37 °C. To initiate proximity-dependent biotinylation, 2 mL of DPBS containing 6 µL of 3% hydrogen peroxide was added directly to each dish (final H_2_O_2_ ≈ 0.9 mM in a total volume of 6 mL). Dishes were swirled rapidly for even coverage and incubated for exactly 45 s at room temperature. The reaction was immediately quenched by adding 6 mL of 2× quenching solution (10 mM Trolox, 20 mM sodium ascorbate, and 20 mM sodium azide in DPBS) and placing dishes on ice for 1 min. Quenching solution was removed, and cells were washed once with 4 mL of 1× quenching solution. After aspiration, dishes were stored at −80 °C until lysis. Negative-control samples were processed identically but without the addition of H_2_O_2_.

### 2.4. Cell Lysis and Streptavidin Enrichment of Biotinylated Proteins

Cells were lysed by adding 0.3 mL of lysis buffer (8 M urea, 1% SDS, 100 mM NH_4_HCO_3_, 10 mM tris(2-carboxyethyl)phosphine (TCEP), and 40 mM sodium phosphate, pH 8.0) directly to each 10 cm dish. Lysates were homogenized by gentle rotation and collected with a cell scraper. Proteins were precipitated by adding 0.3 mL of 55% (*w*/*v*) trichloroacetic acid (TCA), incubating on ice for 15 min, and centrifuging at 21,000× *g* for 10 min at 4 °C. Pellets were washed three times with 1 mL of cold acetone (−30 °C).

For each biological replicate, washed pellets from three 10 cm dishes were pooled and resuspended in a final volume of 0.6 mL lysis buffer and subjected to sonication in an ultrasonic water bath for 1 h. Insoluble material was removed by centrifugation at 21,000× *g* for 10 min at room temperature, and supernatants were transferred to new tubes. Free cysteines were alkylated by adding 40 µL of freshly prepared iodoacetamide (400 mM in 50 mM NH_4_HCO_3_) and incubating for 25 min at room temperature in the dark. Excess iodoacetamide was quenched by adding 45 µL of 1 M dithiothreitol (DTT). Samples were diluted with 685 µL of water to achieve final concentrations of 4 M urea and 0.5% SDS. Three such independent biological replicate preparations were processed per condition.

For streptavidin pull-down, 75 µL of streptavidin magnetic bead suspension (Invitrogen, Carlsbad, CA, USA, Cat. #65602) was washed twice with wash buffer (50 mM sodium phosphate, pH 8.0, 0.4 M urea, and 0.5% SDS). Diluted lysates were incubated with pre-washed beads overnight at 4 °C with end-over-end rotation. Beads were then collected on a magnetic rack and washed three times with wash buffer, followed by three washes with the same buffer lacking SDS. After the first wash of each set, beads were transferred to a fresh tube to minimize carryover. Additional high-stringency washes (including 4 M urea-containing buffers) were performed prior to on-bead digestion for proteomic analysis, as described below. Washed beads were either processed immediately or flash-frozen and stored at −80 °C.

### 2.5. Immunoblot Validation of Biotinylation and Fusion-Protein Expression

For streptavidin-HRP blotting, aliquots of total lysate, unbound fraction, and bead-bound material were mixed with SDS sample buffer, heated, separated by SDS-PAGE, and transferred to a PVDF membrane. Biotinylated proteins were detected using streptavidin conjugated to horseradish peroxidase (streptavidin-HRP; Bio-Rad, Hercules, CA, USA, Cat. #1610381) followed by chemiluminescent development. For fusion-protein expression, whole-cell lysates were immunoblotted using an anti-Nanog antibody (R&D Systems, Minneapolis, MN, USA Cat. #AF1997); GAPDH (MilliporeSigma, Burlington, MA, USA, Cat. #CB1001) was used as a loading control. Antibody dilutions and incubation conditions were used according to the manufacturer’s recommendations.

### 2.6. Fluorescence Microscopy

mApple-APEX-NANOG expression and cellular localization were assessed by fluorescence microscopy. MSCs were induced with doxycycline, counterstained with Hoechst 33342 to visualize nuclei, and imaged using an EVOS fluorescence microscope (Thermo Fisher Scientific, Waltham, MA, USA) equipped with a 60× objective. mApple fluorescence was collected in the Texas Red channel, and Hoechst 33342 fluorescence in the DAPI channel.

### 2.7. Proteomic Sample Preparation, On-Bead Digestion, and TMTpro Labeling

Following streptavidin enrichment, beads were washed three times with 4 M urea, 0.5% SDS, 50 mM sodium phosphate (pH 8.0), three times with the same buffer lacking SDS, and three times with PBS. Washed beads were resuspended in 200 mM EPPS buffer (pH 8.5) containing 2% acetonitrile. On-bead digestion was initiated by adding endoproteinase LysC (FUJIFILM Wako Chemicals U.S.A. Corporation, Richmond, VA, USA) at a 1:50 enzyme-to-protein ratio (*w*/*w*) and incubating for 3 h at 37 °C with shaking. Trypsin (Promega, Madison, WI, USA) was then added at a 1:100 enzyme-to-protein ratio (*w*/*w*), and digestion was continued overnight at 37 °C. Digestion efficiency was assessed by LC-MS using a small aliquot, and samples were required to have a missed-cleavage rate < 10%.

Acetonitrile was added to the digested peptides to a final concentration of 30% (*v*/*v*), and peptides were labeled with TMTpro 18-plex reagents (Thermo Fisher Scientific, Waltham, MA, USA, Cat. #A52045) for 1 h at room temperature with periodic mixing. Labeling efficiency (>95%) was confirmed by LC-MS on a pooled test aliquot. Reactions were quenched with 0.5% hydroxylamine for 15 min and acidified with formic acid. Labeled samples were pooled and concentrated to near dryness by vacuum centrifugation.

### 2.8. High-pH Reversed-Phase Peptide Fractionation

Pooled TMT-labeled peptides were resuspended in 1% formic acid and 0.1% trifluoroacetic acid and fractionated using the Pierce High pH Reversed-Phase Peptide Fractionation Kit (Thermo Fisher Scientific, Waltham, MA, USA, Cat. #84868). A 12-step acetonitrile gradient was used to elute peptides at 10%, 11.75%, 13.5%, 15.25%, 17%, 18.75%, 20.5%, 22.25%, 24%, 25.75%, 27.5%, and 80% acetonitrile. Fractions were dried by vacuum centrifugation, desalted using StageTips, and resuspended in 3% acetonitrile and 1% formic acid for LC-MS analysis.

### 2.9. LC-MS/MS Data Acquisition

Peptides were separated using a Vanquish Neo nano-uHPLC system (Thermo Fisher Scientific) equipped with a 25 cm × 75 µm inner diameter, 1.7 µm C18 Aurora Ultimate capillary column (IonOpticks, Collingwood, VIC, Australia) maintained at 60 °C. Peptides were eluted at 500 nL/min using Buffer A (0.1% formic acid in water) and Buffer B (95% acetonitrile, 0.1% formic acid) over 60 min or 180 min gradients. Analytical gradients were followed by a high-organic (95% acetonitrile) wash and re-equilibration steps prior to the next injection.

Mass spectrometry was performed on an Orbitrap Exploris 480 mass spectrometer (Thermo Fisher Scientific, Waltham, MA, USA) using an MS2-based TMTpro workflow coupled to a FAIMS ProDuo2 interface (compensation voltages −40 V, −60 V, and −80 V). Full MS1 scans were acquired in the Orbitrap at 120,000 resolution, followed by high-energy collisional dissociation (HCD; 33% normalized collision energy). MS2 spectra were acquired in the Orbitrap at 30,000 resolution using a quadrupole isolation window of 0.7 m/z. The maximum injection time was set to 100 ms for MS1 scans, with MS2 injection times ranging from 59 to 400 ms. Quantitative acquisition parameters were based on a previously described workflow [21].

### 2.10. Database Searching and Quantitative Proteomics Analysis

Raw data were searched using Comet against a concatenated target–decoy UniProt reference proteome (downloaded 19 April 2024). Searches were performed with a precursor mass tolerance of 20 ppm and a fragment ion tolerance of 0.02 Da. Static modifications included TMTpro labeling of peptide N-termini and lysine residues (+304.207145 Da) and carbamidomethylation of cysteine residues (+57.0214637 Da); oxidation of methionine residues (+15.994914 Da) was included as a variable modification. Up to two missed tryptic cleavages were allowed.

Peptide-spectrum matches were filtered to a 1% false discovery rate (FDR) using a target–decoy strategy with linear discriminant analysis (LDA), followed by protein assembly to achieve a final protein-level FDR of 1%. TMT reporter ion intensities were extracted using a 0.003 Da window centered on each theoretical reporter m/z and corrected for isotopic impurities according to manufacturer specifications. Protein quantification was performed by summing reporter ion signal-to-noise (S/N) values across all PSMs assigned to each protein. Quantified peptides were required to have a summed TMT S/N > 200 across all channels and an isolation specificity ≥ 70%.

### 2.11. Statistical Analysis and Hit Calling

Protein-level log_2_ fold changes and −log_10_(*p*-values) were provided in the processed volcano summary table generated from the quantitative comparison of three independent biological replicates per condition (*n* = 3 per group), where each biological replicate represented pooled material from three 10 cm dishes. For reporting, *p*-values were obtained by converting the provided −log_10_(*p*) values (*p* = 10^−(−log10p)^). Proteins meeting the prioritization criterion of positive log_2_ fold change (NANOG APEX/control) and nominal *p* < 0.05 were designated APEX-enriched candidates, consistent with the volcano plot coloring. Because the study size is modest and the reported values are nominal *p*-values, this threshold is intended for candidate prioritization and module-level interpretation rather than as sole evidence of direct physical interaction.

### 2.12. Literature Cross-Check and Background Assessment

Significant APEX-enriched proteins were cross-referenced against curated NANOG/Nanog interaction records in BioGRID [22] to identify previously reported NANOG/Nanog-linked interaction evidence. Supplementary Table S1 now reports the supporting reference number together with the evidence class and study context (species, cell type, and technique) for curated prior records where available; blank entries indicate that no prior curated record was retrieved in this cross-check. Background propensity was assessed using CRAPome [23] contaminant classes as a heuristic flag; because CRAPome [23] is largely derived from affinity purification–mass spectrometry (AP-MS) experiments, these flags should be interpreted as background-prone categories rather than definitive exclusions for proximity labeling.

### 2.13. Unbiased Enrichment Analysis and Module Prioritization

Unbiased over-representation analysis was performed on the 828 APEX-enriched proteins using GO Biological Process, Reactome, and KEGG human gene-set collections, with the human gene universe represented in each library used as background. For each term, significance was calculated with a hypergeometric test and adjusted for multiple testing by the Benjamini–Hochberg method. Terms with FDR < 0.05 were considered significant. Detailed results are provided in Supplementary Tables S2–S4. The modules highlighted in the main text were not selected only by raw *p*-value ranking. Instead, they were chosen as representative submodules nested within the significant enrichment families when they were supported by at least one FDR-significant enrichment term, contained multiple APEX-enriched proteins, and described coherent nuclear or MSC-relevant biology.

## 3. Results and Discussion

**Figure 1:**
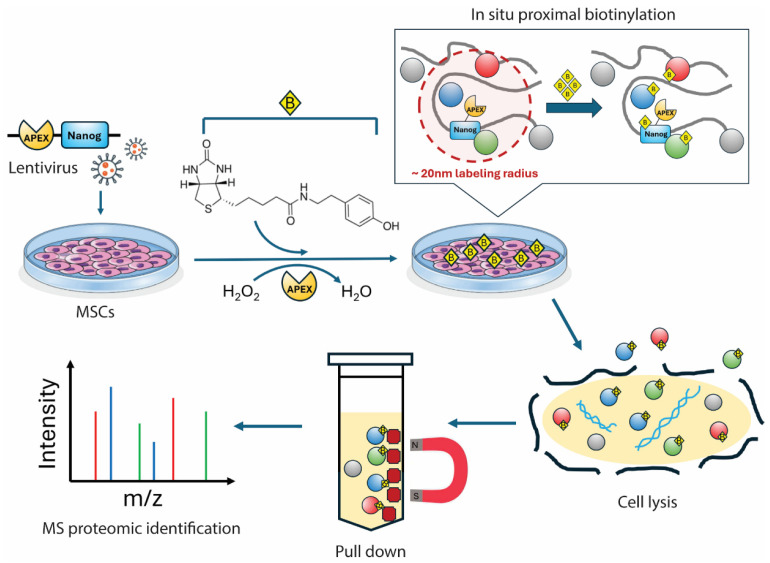
APEX-mediated proximity biotinylation workflow for mapping the NANOG proximal proteome in mesenchymal stem cells (MSCs). MSCs were transduced with a doxycycline-inducible lentiviral construct encoding an in-frame mApple-APEX-NANOG fusion protein. After induction, cells were incubated with the peroxidase substrate biotin-tyramide (diamond symbols) and briefly exposed to hydrogen peroxide (H_2_O_2_) to activate APEX-catalyzed formation of short-lived phenoxyl radicals. This reaction results in covalent biotinylation of proteins in the immediate vicinity of NANOG in living cells (~20 nm labeling radius, as indicated in the schematic). Cells were then quenched and lysed, and biotinylated proteins were enriched by streptavidin affinity capture prior to identification by liquid chromatography-tandem mass spectrometry (LC-MS/MS).

**Figure 2:**
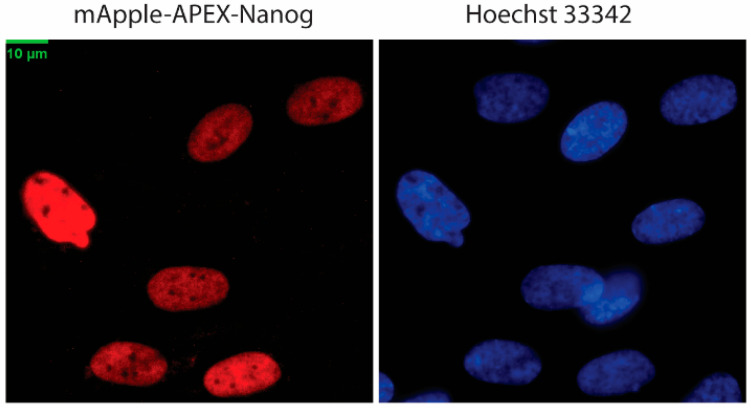
Subcellular localization of mApple-APEX-NANOG in MSCs. Representative fluorescence micrographs of MSCs expressing mApple-APEX-NANOG (red; Texas Red channel) with nuclei counterstained with Hoechst 33342 (blue; DAPI channel). Images were acquired on an EVOS fluorescence microscope using a 60× objective. Scale bar, 10 µm.

**Figure 3:**
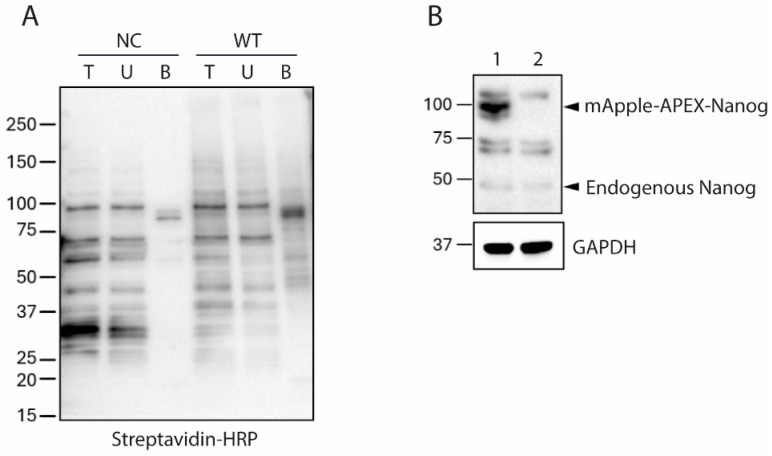
Validation of APEX-mediated biotinylation and fusion-protein expression in MSCs. (**A**) Streptavidin-horseradish peroxidase (HRP) immunoblot of total lysate (T), unbound fraction (U), and streptavidin bead-bound fraction (**B**) from MSCs processed for proximity labeling. The NC negative control condition was processed identically but without H_2_O_2_, whereas the WT labeling condition received H_2_O_2_ for 45 s to initiate APEX-catalyzed biotinylation. The original streptavidin-horseradish peroxidase (HRP) blot image corresponding to (**A**) can be found in Supplementary Figure S1. (**B**) Immunoblot using an anti-NANOG antibody showing expression of the mApple-APEX-NANOG fusion protein in transduced MSCs (lane 1) compared with untransduced parental MSCs (lane 2), alongside endogenous NANOG species. GAPDH served as a loading control. The original Western blot images corresponding to (**B**) can be found in Supplementary Figure S2.

The APEX workflow used to map the NANOG proximal proteome is outlined in Figure 1. MSCs expressing the mApple-APEX-NANOG fusion protein were incubated with biotin-tyramide and briefly exposed to H_2_O_2_ to trigger APEX-dependent biotinylation, which was then quenched. Nuclear localization of the fusion protein was confirmed by fluorescence microscopy (Figure 2), and APEX-dependent biotinylation together with fusion-protein expression was validated by immunoblotting (Figure 3). Biotinylated proteins were enriched using streptavidin, digested, TMT-labeled, and quantified by LC-MS/MS (see Section 2). Comparing +H_2_O_2_ labeling versus the −H_2_O_2_ control defined a NANOG-centered proximity proteome in live MSCs.

### 3.1. Global Features of the +H_2_O_2_ Versus −H_2_O_2_ NANOG Proximity Proteome

**Figure 4:**
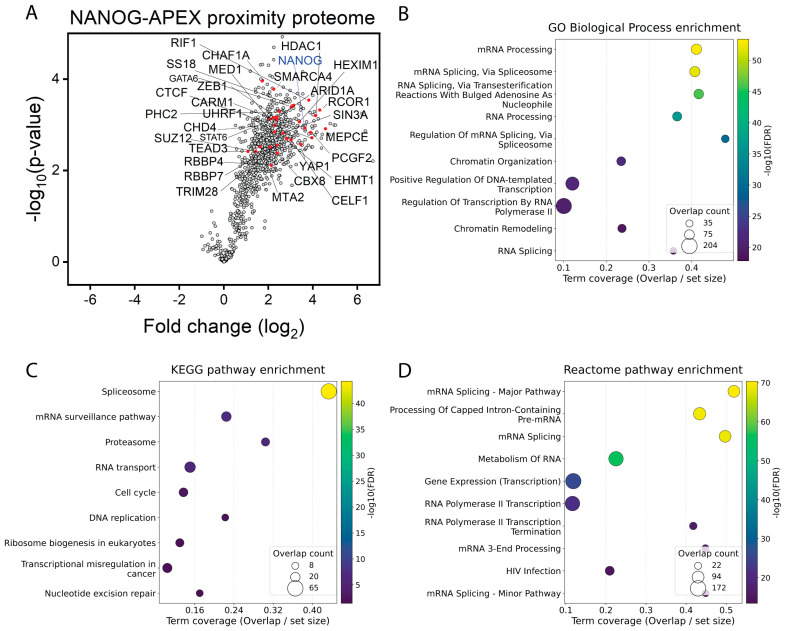
Global features of the NANOG-APEX proteome and unbiased enrichment profile. (**A**) Volcano plot summarizing proteins quantified by mass spectrometry following APEX-based proximity labeling with NANOG–APEX (+H_2_O_2_) compared with the −H_2_O_2_ control. The x-axis shows the log_2_ fold change (+H_2_O_2_/−H_2_O_2_) and the y-axis shows statistical significance as −log_10_(*p*-value). Each point represents one protein. Proteins meeting the enrichment criterion (positive log_2_ fold change and nominal *p* < 0.05) are highlighted in red; all other quantified proteins are shown as open circles. The bait protein NANOG is indicated in blue. Proteins annotated in the plot are representative and are discussed in the module summaries (Figure 5; Table 1) and in Supplementary Table S1. Samples were analyzed from three independent biological replicates per condition; each replicate comprised three 10 cm dishes pooled to provide sufficient material before streptavidin enrichment and LC-MS/MS. (**B**–**D**) Dot plots summarizing representative same-level enrichment terms from unbiased over-representation analysis of the full 828-protein APEX-enriched set for GO Biological Process (**B**), KEGG (**C**), and Reactome (**D**). The x-axis indicates term coverage (Overlap/set size), dot size indicates overlap count, and color indicates −log_10_(FDR). Fully ranked enrichment results are provided in Supplementary Tables S2–S4.

As summarized in Figure 4A, the +H_2_O_2_ versus −H_2_O_2_ comparison yielded 1040 quantified proteins (Supplementary Table S1). The volcano plot was strongly shifted toward positive log_2_ fold changes, consistent with APEX-dependent labeling around NANOG. Using positive log_2_ fold change and nominal *p* < 0.05 as the candidate threshold, 828 proteins were classified as APEX-enriched. Because the study includes three biological replicates per condition and uses nominal *p*-values, we treat this list as a prioritized neighborhood set rather than a list of proven direct binders. Cross-checking against curated NANOG interaction resources identified prior NANOG-linked evidence for 114 proteins, whereas 714 proteins lacked prior support in those resources and may represent MSC-context or previously unreported proximity neighbors.

NANOG itself was strongly enriched (log_2_ fold change ~3.65; *p* ~0.0012) and was supported by multiple quantified peptides. This provides an internal positive control for bait recovery and supports using the broader enriched set for neighborhood-level interpretation.

**Table 1:** Selected illustrative NANOG APEX hits grouped into mechanistically informative submodules within the broader unbiased enrichment landscape. The final column summarizes prior curated NANOG/Nanog-linked evidence where available, including the supporting technique/cell context and manuscript reference number; Detailed protein-level evidence annotations are provided in Supplementary Table S1.

GeneSymbol	UniprotID	log2FC	−log10 (*p*-Value)	*p*-Value	NumPeps Quantified	Technique (Cell Type; Ref)
Transcriptional regulation and co-transcriptional RNA processing						
HEXIM2	Q96MH2	1.42	3.78	0.0002	1	
POLR2C	P19387	2.12	3.17	0.0007	1	
MED1	Q15648	2.07	3.12	0.0008	2	
HEXIM1	O94992	3.40	3.06	0.0009	3	
SUPT5H	O00267	2.45	3.02	0.0010	25	
MEPCE	Q7L2J0	4.57	2.91	0.0012	1	
AFF4	Q9UHB7	2.59	2.90	0.0012	1	
NELFA	Q9H3P2	2.39	2.82	0.0015	3	
CDK9	P50750	1.52	2.79	0.0016	1	
NELFE	P18615	2.59	2.74	0.0018	2	
ELOA	Q14241	1.93	2.73	0.0019	5	
POLR2A	P24928	2.20	2.70	0.0020	40	AP-MS (mESC; 47)
MED8	Q96G25	1.49	2.52	0.0030	2	
MED12	Q93074	3.68	2.48	0.0033	1	targeted interaction/ChIP (ESC; 51)
POLR2D	O15514	2.89	2.28	0.0053	2	
LARP7	Q4G0J3	1.40	2.22	0.0060	2	
POLR2B	P30876	1.37	2.20	0.0063	14	AP-MS (mESC; 47)
SUPT6H	Q7KZ85	1.24	2.16	0.0069	6	
SUPT16H	Q9Y5B9	1.69	2.13	0.0075	13	AP-MS/co-IP (hNT2; 48)
ELOB	Q15370	1.28	2.07	0.0086	7	
SSRP1	Q08945	2.20	2.03	0.0093	3	AP-MS/ChIP-seq (ESC; 37)
NELFCD	Q8IXH7	1.28	1.91	0.0122	3	
ELL	P55199	0.73	1.82	0.0153	1	
PHAX	Q9H814	5.02	2.99	0.0010	1	
PRPF39	Q86UA1	4.01	3.22	0.0006	3	
PLRG1	O43660	4.05	3.08	0.0008	3	
RBM27	Q9P2N5	4.00	3.64	0.0002	1	
DAZAP1	Q96EP5	4.74	2.78	0.0017	4	
ALKBH5	Q6P6C2	2.27	2.84	0.0015	2	
METTL14	Q9HCE5	2.06	2.68	0.0021	1	
WTAP	Q15007	2.01	2.59	0.0026	4	co-IP/MS (hPSC; 52)
YTHDC1	Q96MU7	1.92	2.03	0.0094	4	
PRC1/PRC2 (chromatin repression/compaction)						
CBX3	Q13185	2.68	3.49	0.0003	2	AP-MS (mESC; 47)
PHC2	Q8IXK0	2.49	3.29	0.0005	2	
YY1	P25490	3.25	2.94	0.0012	3	
CBX5	P45973	3.53	2.94	0.0012	7	
PCGF2	P35227	3.90	2.83	0.0015	1	AP-MS/co-IP (hNT2; 48)
RBBP7	Q16576	2.09	2.51	0.0031	3	affinity capture/MS (mESC; 11)
CBX1	P83916	1.72	2.42	0.0038	2	AP-MS/ChIP-seq (ESC; 37)
SUZ12	Q15022	1.44	2.42	0.0038	1	AP-MS/co-IP (hNT2; 48)
CBX8	Q9HC52	2.40	2.38	0.0042	1	
SCML2	Q9UQR0	2.12	2.27	0.0054	1	
RBBP4	Q09028	2.14	2.11	0.0077	3	AP-MS/ChIP-seq (ESC; 37)
3D genome and replication-coupled chromatin						
CHAF1A	Q13111	1.73	3.96	0.0001	1	
RIF1	Q5UIP0	3.82	3.54	0.0003	4	AP-MS (mESC; 49); affinity capture/MS (mESC; 11); AP-MS/ChIP-seq (ESC; 37)
MCMBP	Q9BTE3	3.88	3.26	0.0005	1	
WRNIP1	Q96S55	3.66	2.90	0.0013	2	
UHRF1	Q96T88	2.28	2.84	0.0014	2	AP-MS/ChIP-seq (ESC; 37)
RAD21	O60216	2.64	2.78	0.0017	4	co-localization (mESC; 37)
CTCF	P49711	3.96	2.71	0.0019	1	AP-MS/ChIP-seq (ESC; 37)
WAPL	Q7Z5K2	2.29	2.71	0.0019	2	AP-MS/ChIP-seq (ESC; 37)
MCM5	P33992	2.59	2.67	0.0022	11	
MCM2	P49736	1.92	2.47	0.0034	4	AP-MS/co-IP (hNT2; 48)
NIPBL	Q6KC79	1.54	2.35	0.0045	3	
MCM3	P25205	2.63	2.24	0.0057	10	AP-MS/ChIP-seq (ESC; 37)
MCM6	Q14566	2.35	2.17	0.0068	4	
ORC2	Q13416	0.97	1.74	0.0183	1	
NuRD/HDAC repression (deacetylation/remodeling)						
SUDS3	Q9H7L9	2.78	3.57	0.0003	1	
RCOR1	Q9UKL0	4.31	3.32	0.0005	1	
HDAC1	Q13547	4.13	3.20	0.0006	1	affinity capture/MS (mESC; 11); AP-MS/ChIP-seq (ESC; 37)
RCOR3	Q9P2K3	2.15	3.08	0.0008	4	
CHD4	Q14839	2.29	2.99	0.0010	8	AP-MS/ChIP-seq (ESC; 37); AP-MS (mESC; 47)
SAP30BP	Q9UHR5	3.32	2.98	0.0011	4	
SIN3A	Q96ST3	2.66	2.81	0.0015	2	AP-MS/co-IP (hNT2; 48); affinity capture/MS (mESC; 11); AP-MS (mESC; 47)
NCOR2	Q9Y618	3.15	2.77	0.0017	7	AP-MS/co-IP (hNT2; 48); quantitative AP-MS (mESC; 50)
MTA3	Q9BTC8	1.78	2.64	0.0023	1	AP-MS/co-IP (hNT2; 48); quantitative AP-MS (mESC; 50); AP-MS (mESC; 47)
TRIM28	Q13263	2.40	2.55	0.0028	21	AP-MS (mESC; 49); affinity capture/MS (mESC; 11); AP-MS/ChIP-seq (ESC; 37)
MTA2	O94776	1.63	2.52	0.0031	3	AP-MS/co-IP (hNT2; 48); affinity capture/MS (mESC; 11); AP-MS/ChIP-seq (ESC; 37); quantitative AP-MS (mESC; 50); AP-MS (mESC; 47)
GATAD2A	Q86YP4	1.33	2.25	0.0056	1	affinity capture/MS (mESC; 11); quantitative AP-MS (mESC; 50); AP-MS (mESC; 47)
HDAC2	Q92769	1.63	2.23	0.0059	5	AP-MS (mESC; 49); affinity capture/MS (mESC; 11); quantitative AP-MS (mESC; 50)
NCOR1	O75376	0.84	1.97	0.0107	3	AP-MS (mESC; 47)
GATAD2B	Q8WXI9	0.84	1.82	0.0153	2	affinity capture/MS (mESC; 11); AP-MS (mESC; 47)
BAF (SWI/SNF) chromatin remodeling						
SMARCA4	P51532	2.25	3.78	0.0002	4	affinity capture/MS (mESC; 11)
SS18	Q15532	3.14	3.43	0.0004	4	
ARID1B	Q8NFD5	2.17	3.38	0.0004	7	AP-MS/co-IP (hNT2; 48)
ARID1A	O14497	2.40	3.16	0.0007	18	AP-MS (mESC; 47)
SMARCC1	Q92922	2.90	2.82	0.0015	6	
ACTL6A	O96019	2.14	2.66	0.0022	4	
DPF2	Q92785	1.98	2.64	0.0023	2	AP-MS (mESC; 47)
BRD7	Q9NPI1	2.14	2.61	0.0024	1	
SMARCE1	Q969G3	3.11	2.50	0.0032	1	
SMARCA2	P51531	0.92	1.76	0.0172	2	affinity capture/MS (mESC; 11)
Mechanical and growth factor signaling/mesenchymal identity						
CEBPB	P17676	1.73	3.97	0.0001	1	
LOX	P28300	3.55	3.67	0.0002	4	
FOSL2	P15408	2.37	3.65	0.0002	6	
SPARC	P09486	1.26	3.48	0.0003	1	
GATA6	Q92908	3.03	3.39	0.0004	2	
DKK1	O94907	1.25	3.27	0.0005	1	
FOSL1	P15407	2.04	3.18	0.0007	5	
ZEB1	P37275	2.25	3.15	0.0007	4	
STAT6	P42226	2.06	3.12	0.0008	2	
JUND	P17535	3.38	3.09	0.0008	3	
FGF2	P09038	2.47	3.08	0.0008	1	
JUNB	P17275	1.93	3.06	0.0009	6	
STAT5A	P42229	2.61	2.96	0.0011	1	
YAP1	P46937	3.46	2.57	0.0027	5	
SMAD2	Q15796	1.99	2.43	0.0037	3	
TEAD3	Q99594	1.08	2.41	0.0038	4	
VTN	P04004	2.23	2.29	0.0051	1	
TWIST1	Q15672	1.88	2.01	0.0097	1	
FOS	P01100	1.32	1.97	0.0108	4	
FOSB	P53539	1.11	1.94	0.0114	1	
VIM	P08670	0.58	1.85	0.0140	5	
IGFBP3	P17936	0.96	1.62	0.0238	1	
FN1	P02751	1.50	1.60	0.0250	5	
MIA3	Q5JRA6	1.91	1.58	0.0265	1	
CCN1	O00622	1.32	1.48	0.0330	4	
COL3A1	P02461	0.95	1.44	0.0359	9	
TIMP3	P35625	0.72	1.38	0.0413	1	

**Figure 5:**
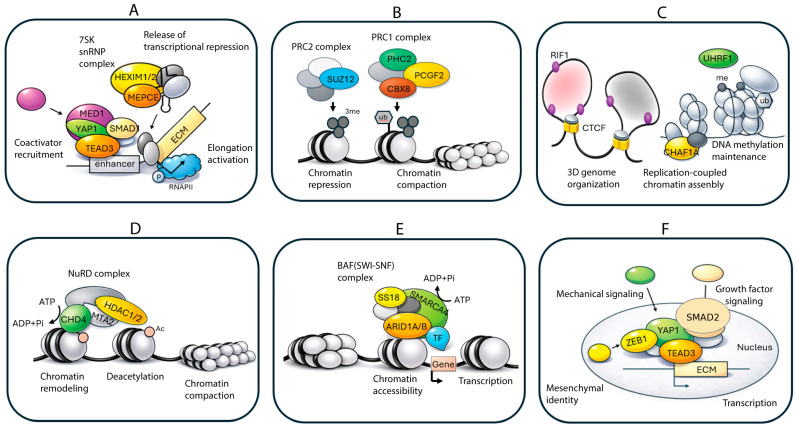
Functional modules represented within the APEX-NANOG proxeome. Schematic overview of selected proteins and pathways represented among proteins captured by APEX-NANOG proximity labeling in MSCs and identified by LC-MS/MS. Representative proteins are indicated within each module: (**A**) transcriptional regulation and co-transcriptional RNA processing, including 7SK/P-TEFb and mediator-linked elongation control together with spliceosomal/RNP and selected m^6^A-related factors (MEPCE, HEXIM1/2, MED1, PHAX, PRPF39, ALKBH5, METTL14, WTAP, YTHDC1); (**B**) Polycomb repressive complexes PRC1/PRC2 (PCGF2, CBX8, PHC2, SUZ12) associated with chromatin repression and compaction; (**C**) genome architecture and replication-coupled chromatin regulation factors (CTCF, RIF1, CHAF1A, UHRF1); (**D**) NuRD chromatin remodeling/deacetylation complex components (HDAC1/2, CHD4, MTA2); (**E**) BAF (SWI/SNF) chromatin remodeling complex components (SMARCA4, ARID1A/B, SS18); and (**F**) mechanosensitive and growth factor signaling transcriptional regulators (ZEB1, YAP1, TEAD3, SMAD2).

### 3.2. RNA Processing/Splicing, Transcriptional Elongation, and m^6^A-Related RNA Metabolism

We also analyzed the full 828-protein hit list by unbiased over-representation analysis, summarized in Figure 4B–D and detailed in Supplementary Tables S2–S4. Across GO Biological Process, Reactome, and KEGG, RNA processing and pre-mRNA splicing were the strongest and most consistent signals. GO terms included mRNA processing (88/214 proteins; FDR = 3.95 × 10^−54^) and mRNA splicing via spliceosome (86/211; FDR = 9.93 × 10^−53^). Reactome highlighted mRNA splicing-major pathway (94/181; FDR = 4.15 × 10^−71^) and processing of capped intron-containing pre-mRNA (105/242; FDR = 5.45 × 10^−70^), while KEGG identified the spliceosome (65/150; FDR = 5.54 × 10^−45^) as the top pathway. This convergence likely reflects a genuine feature of the NANOG-proximal nuclear neighborhood rather than a single-pathway artifact. Because APEX reports local spatial neighborhoods rather than only direct binary interactions, recovery of splicing and RNP factors is biologically plausible if NANOG occupies transcriptionally active or poised chromatin domains where RNA polymerase II elongation and co-transcriptional splicing are coordinated [24]. This interpretation is also consistent with prior studies showing that Nanog itself is alternatively spliced into isoforms with different self-renewal activities, that SRSF3 promotes pluripotency in part through Nanog mRNA export, and that the splicing regulator SON supports accurate processing of pluripotency-associated transcripts in human embryonic stem cells [25,26,27]. In MSC-related systems, this RNA-centered interpretation is likewise plausible because NANOG overexpression improves proliferative and functional phenotypes in aging MSC models [5,6], whereas post-transcriptional control has emerged as an important determinant of bone marrow stromal cell fate, aging, and osteogenic competence, including YBX1-dependent splicing homeostasis and METTL3-dependent m6A and alternative-splicing regulation [28,29,30]. Together, these observations support the view that NANOG-associated nuclear hubs in MSCs may coordinate chromatin regulation with transcript maturation and RNA fate decisions relevant to stemness and mesenchymal plasticity.

Consistent with this dominant RNA-centered enrichment, the NANOG WT proximity proteome also contained multiple regulators of transcriptional elongation, pause release, and associated RNA-processing machinery (Figure 5A; Table 1). MEPCE, the methylphosphate capping enzyme for 7SK snRNA, displayed a high log_2_ fold-change (~4.57), and LARP7 and HEXIM1/HEXIM2 were also enriched, together with CDK9 and AFF4. These proteins regulate sequestration and release of P-TEFb, which controls RNA polymerase II pause-release and productive elongation [31,32]. The co-enrichment of 7SK/P-TEFb components with RNA polymerase II subunits (e.g., POLR2A/B/C), Mediator components (MED1, MED12), spliceosomal/RNP factors (PHAX, PRPF39, PLRG1, RBM27, DAZAP1), and selected m^6^A-linked regulators (ALKBH5, METTL14, WTAP, YTHDC1) suggests that NANOG-associated nuclear environments include a coupled transcription–RNA processing axis characteristic of dynamic transcriptional hubs.

The enriched set also contains RNA modification factors, including METTL14 and WTAP (known partners of the m^6^A writer complex), as well as the m^6^A demethylase ALKBH5 and nuclear reader YTHDC1. m^6^A RNA modification has been shown to regulate pluripotency and differentiation in both mouse and human ESCs, with mapping studies revealing conserved m^6^A decoration on transcripts encoding core pluripotency transcription factors such as NANOG [33]. Notably, YTHDC1 can directly couple nuclear m^6^A recognition to alternative splicing regulation [34]. The presence of m^6^A writers, erasers, and readers in the NANOG proxeome therefore implies that NANOG-centered chromatin hubs are coupled to co-transcriptional RNA regulation, potentially tuning the stability, processing, and export of transcripts associated with stemness and mesenchymal plasticity.

### 3.3. Enrichment of Polycomb and HDAC/NuRD/NODE Repression Machinery

Beyond the dominant RNA-processing signal, a pronounced Polycomb signature was evident in the NANOG proximity proteome. Polycomb group proteins associated with PRC1, including PCGF2, CBX8, and PHC2, were significantly enriched, with log_2_ fold-changes of approximately 3.9, 2.4, and 2.5, respectively. The PRC2 core subunit SUZ12 was also enriched, along with the histone-binding proteins RBBP4 and RBBP7, which can participate in both PRC2 and NuRD complexes. Polycomb complexes play central roles in repressing developmental regulators while maintaining them in a poised state in human embryonic stem cells [35]. The recovery of these factors in the NANOG APEX dataset suggests that NANOG is proximal to Polycomb-associated repression and poising machinery in the MSC-like context studied here.

In parallel, we observed strong enrichment of histone deacetylase and NuRD/NODE-related proteins. HDAC1 exhibited one of the highest enrichments in the dataset, with a log_2_ fold-change of approximately 4.13 and *p* ~6 × 10^−4^. HDAC2, CHD4 (the NuRD ATPase), and MTA2/MTA3 were similarly enriched, as were SIN3A, SUDS3, and the co-repressor RCOR1 (CoREST) (Table 1 and Supplementary Table S1). This profile closely resembles the Nanog-Oct4-associated deacetylase (NODE) complex originally described in mESCs, which contains HDAC1/2 and MTA proteins and is linked to the control of ES cell fate decisions [11,12]. The convergence of Polycomb and HDAC/NuRD/NODE components (Figure 5B,D) around NANOG in this proximity proteome strongly suggests that NANOG in MSC-like cells is situated within a repression- and poising-oriented chromatin landscape, echoing but not necessarily identical to the repressor associations seen in mouse ESCs.

### 3.4. Chromatin Remodeling, Three-Dimensional Genome Architecture, and DNA Replication/Repair in the NANOG Neighborhood

Beyond repressive modules, the dataset revealed robust enrichment of ATP-dependent chromatin remodelers (Figure 5C,E; Table 1) of the SWI/SNF (BAF) family. SMARCA4 (BRG1), the core ATPase of many BAF complexes, showed a log_2_ fold-change of about 2.25 and strong statistical support, while ARID1A, ARID1B, SMARCC1, SMARCE1, SS18, ACTL6A, and DPF2 were also enriched. In mouse ESCs, esBAF complexes (BAF assemblies with specific subunit compositions) are essential components of the core pluripotency network, and their genome-wide occupancy overlaps with pluripotency transcription factors and Stat3/Smad signaling [13]. Notably, the concurrent enrichment of both ARID1A (log2 FC 2.40) and ARID1B (log2 FC 2.17) reflects a versatile BAF composition consistent with an MSC-like identity, distinguishing it from the ARID1A-dominant esBAF profile typically found in pluripotent cells. The enriched SWI/SNF modules in the current dataset indicate that NANOG in MSC-like cells is in close proximity to chromatin remodeling machinery capable of modulating nucleosome positioning and accessibility at regulatory elements.

We also noted significant enrichment of genome architecture factors, including CTCF and cohesin-associated proteins such as RAD21, WAPL, and NIPBL, along with replication-timing and epigenetic-maintenance factors such as RIF1, UHRF1, and CHAF1A. Additional replication-associated proteins included MCMBP, WRNIP1, and MCM2/3/5/6, consistent with the unbiased Reactome and KEGG enrichment for DNA replication, DNA replication pre-initiation, and DNA repair (Supplementary Tables S2–S4). Cohesin component RAD21 has previously been shown in mESCs to cooperate with pluripotency transcription factors, including Nanog, Oct4, and Sox2, and to play a role in maintaining ESC identity by supporting a specific pattern of cohesin binding at pluripotency-associated loci [36]. CTCF, which acts as a chromatin insulator and loop anchor, has been reported to be important for human ESC proliferation and to associate with pluripotency genes, including NANOG [37]. The predominance of the cohesin unloader WAPL (log_2_ FC 2.29) over the loader NIPBL (log_2_ FC 1.54) suggests a high-turnover state of chromatin loops. This dynamic architectural environment is characteristic of lineage-primed MSCs, facilitating the flexible genomic reconfiguration required for differentiation. The combined enrichment of BAF remodelers, CTCF, cohesin, and replication-timing factors in the NANOG APEX dataset thus supports a model in which NANOG is proximally linked to higher-order chromatin architecture and dynamic chromatin remodeling, not merely local transcription factor binding. Consistent with the broad architectural neighborhood captured by APEX, human NANOG can form prion-like assemblies that bridge DNA and reorganize chromatin [14], which could help concentrate chromatin remodelers and genome-architecture factors within the NANOG-centered labeling radius.

### 3.5. MSC-Related Signaling Regulators and Other Significant Non-Chromatin Hits

Beyond chromatin and transcription, the dataset recovered several MSC-relevant signaling and transcriptional regulators (Figure 5F; Table 1). YAP1, a central effector of mechanotransduction and Hippo signaling, was strongly enriched, as was TEAD3, a DNA-binding partner for YAP/TAZ. SMAD2, which functions downstream of TGFβ/Activin signaling, was enriched alongside mesenchymal and epithelial–mesenchymal transition (EMT)-associated transcription factors ZEB1 and TWIST1, and lineage-priming factor CEBPB. These proteins are well-aligned with known pathways controlling MSC differentiation, mechanosensitivity, and immunomodulatory output [3,18,38]. Their presence in the NANOG APEX proxeome suggests that NANOG in MSC-like cells operates at the crossroads of chromatin regulation and signal-responsive transcription, potentially integrating mechanical and growth factor cues into nuclear regulatory programs.

Several proteins linked to MSC identity and signal integration were enriched in the NANOG proxeome, including mechanotransduction and TGFβ-linked transcriptional regulators. While many of these proteins lack prior direct interaction evidence with NANOG in curated databases, their recovery is unlikely to be explained solely by non-specific background because they are functionally coherent as a group; they include nuclear transcriptional regulators rather than dominant AP-MS contaminants, and their biology is directly relevant to MSC fate control and aging-associated drift. We therefore treat this subset as MSC-context hypotheses, highlighting that NANOG proxeome mapping can recover mesenchymal signaling neighborhoods that are not prominent in ESC-centered NANOG interaction maps. Although this signaling subset does not define the highest-ranked global enrichment terms, we retain it because it addresses the central biological question of how NANOG-associated nuclear neighborhoods may interface with mesenchymal signaling and fate control.

Mechanistically, there is direct literature precedent linking NANOG to the same signaling axes recovered by APEX. YAP1/TAZ-TEAD activity is a core mechanotransduction module that is required for stiffness-dependent fate specification in mesenchymal stem/stromal cells, and TEAD-dependent Hippo output has been shown to impinge on the NANOG circuitry (including TEAD-mediated regulation of NANOG promoter activity in stem-cell contexts), providing a plausible route by which mechanical cues could converge on NANOG-associated nuclear programs [39,40]. In parallel, TGFβ/Activin/Nodal signaling is directly wired to NANOG through SMAD2/3: SMAD2/3 occupy functional SMAD-response elements at the NANOG promoter, and Activin/Nodal signaling sustains NANOG expression, offering a straightforward explanation for why SMAD2 was recovered within the NANOG proximity environment [41,42]. Given that Hippo effectors can also cooperate with SMAD2/3 to tune TGFβ-family transcriptional outputs, the combined enrichment of YAP1/TEAD3 and SMAD2 in the NANOG-APEX proteome supports the idea that NANOG in MSC-like cells sits at a signal-responsive nuclear hub, positioned to integrate mechanotransductive and growth factor cues with EMT/lineage-priming transcriptional modules to shape differentiation trajectories and immunomodulatory state.

We also examined the top significantly enriched proteins after excluding obvious chromatin/histone/ribosomal/keratin/metabolic keywords from annotations to identify “non-chromatin” candidates that might reveal additional biology or proximity-labeling artifacts. This subset included NR2F6, BAG4, PHAX, FOXK1, BCAT1, RBM27, NFIX, RCOR1, RIF1, MT1B, MT2A, QRICH1, DAZAP1, LOX, RFTN1, PRPF39, MCMBP, PLRG1, and ZFAND3. NR2F6 is an orphan nuclear receptor known to regulate T cell activation and function as an intracellular immune checkpoint; its enrichment raises the possibility that NANOG-proximal nuclear environments intersect with nuclear receptors that influence immune-related gene expression [43,44]. PHAX is a factor involved in RNA export, while PRPF39, PLRG1, RBM27, and DAZAP1 participate in pre-mRNA processing and splicing, consistent with a broader proximity of NANOG to transcription-splicing hubs. MCMBP is a replication-associated protein, and LOX (lysyl oxidase) is classically involved in extracellular matrix cross-linking, suggesting that some LOX signal might reflect secreted or matrix-associated proteins that are nonetheless recovered in nuclear-enriched or streptavidin-enriched fractions, or that LOX may have less appreciated nuclear roles.

While some of these non-chromatin hits likely reflect true proximity (especially RNA-processing and replication factors), others may be enriched due to secondary effects of NANOG expression, changes in nuclear organization, or labeling-related stress. Metallothioneins such as MT1B and MT2A, for example, are redox-responsive and can be induced under oxidative conditions, which may be relevant given the H_2_O_2_-dependent chemistry of APEX labeling. Consequently, these non-chromatin hits are best viewed as hypotheses for further validation rather than definitive functional NANOG interactors. A module-based summary and highlighted hits are provided in Table 1, and full protein-level annotations are provided in Supplementary Table S1. Several mechanotransduction and mesenchymal identity regulators are highlighted as Module F in Figure 5 and summarized in Table 1.

A central motivation for mapping the NANOG proxeome in MSCs is that MSC rejuvenation by NANOG overexpression has been demonstrated in aging models, yet the intermediate molecular machinery remains unclear [5,6]. MSC identity and function are shaped by mechanotransduction and soluble-factor signaling (including Hippo/YAP-TEAD and TGFβ/SMAD pathways), which are also implicated in aging-associated changes in lineage bias and immunomodulatory output [3,4]. The recovery of these signal-responsive regulators in the NANOG proxeome suggests that NANOG’s nuclear neighborhood is positioned to integrate extracellular cues with chromatin-based control.

### 3.6. Limitations and Future Directions

A key limitation is the biological context and expression system. The proxeome was generated in human MSCs (ATCC SCRC-4000) maintained under continuous selection to preserve hTERT expression (G418), which may not fully reflect primary, donor-derived MSC biology or differentiation-state heterogeneity. In addition, NANOG was expressed as a lentivirally integrated, doxycycline-inducible mApple-APEX-NANOG fusion; even when nuclear localization is verified, fusion tagging and ectopic expression can alter NANOG abundance, chromatin binding dynamics, and downstream transcriptional state. Because much of the foundational NANOG interaction literature derives from mouse ESCs [7,8,45], and human NANOG has distinct assembly and stability features [14,15,16], direct comparisons across species and cell states will be important for interpreting which neighborhoods are conserved. Additional NANOG interaction and pluripotency-network studies used for contextual comparison are cited in Refs. [46,47,48,49,50,51].

Methodologically, APEX reports spatial proximity rather than direct binding (approximately a 20 nm labeling neighborhood), so identified proteins may represent co-localized nuclear or chromatin microenvironments rather than bona fide NANOG interactors. Background labeling and enrichment artifacts can persist despite the study’s negative controls, and the biotin-tyramide/H_2_O_2_ pulse may transiently perturb redox-sensitive associations. Accordingly, candidate hits are best interpreted as a NANOG-proximal neighborhood in MSC nuclei and will be prioritized for orthogonal validation.

## 4. Conclusions

The NANOG +H_2_O_2_ versus −H_2_O_2_ APEX proxeome in human MSCs indicates that NANOG resides within nuclear neighborhoods enriched for transcriptional regulation and co-transcriptional RNA-processing systems (7SK/P-TEFb regulators, Mediator, RNA polymerase II-associated factors, spliceosomal/RNP proteins, and selected m^6^A-related regulators), together with chromatin repression and poising machinery (Polycomb and HDAC/NuRD/CoREST/SIN3), ATP-dependent chromatin remodeling (BAF/SWI–SNF), and genome architecture and replication-coupled chromatin maintenance (CTCF/cohesin, RIF1, CHAF1A, UHRF1). The proxeome additionally contains MSC-relevant signal-integration factors, suggesting that NANOG-associated neighborhoods interface with mechanotransduction and growth factor-responsive transcription in a mesenchymal context. Collectively, these data provide a spatial proteomic resource and generate testable mechanistic hypotheses for how NANOG expression might influence RNA processing, chromatin state, transcriptional competency, and signal responsiveness in MSCs.

## Data Availability

The original contributions presented in this study are included in the article/Supplementary Materials. Further inquiries can be directed to the corresponding author(s). The processed proteomics summary dataset analyzed in this study is available from the corresponding author upon reasonable request. The raw and processed mass spectrometry data will be deposited in a public proteomics repository prior to publication, and the accession number will be added once assigned.

## Supplementary Materials

The following supporting information can be downloaded at: https://www.mdpi.com/article/10.3390/biom16040531/s1. Figure S1: Original streptavidin-horseradish peroxidase (HRP) blot image corresponding to Figure 3A; Figure S2: Original Western blot images corresponding to Figure 3B; Table S1: Processed APEX-NANOG proximity-proteomics summary dataset; Table S2: Full GO Biological Process over-representation analysis; Table S3: Full Reactome over-representation analysis; Table S4: Full KEGG over-representation analysis.

## Abbreviations

APEX	engineered ascorbate peroxidase
MSCs	mesenchymal stem/stromal cells
proxeome	proximity interactome
NuRD	nucleosome remodeling and deacetylase
PRC	Polycomb repressive complex
BAF	SWI/SNF chromatin remodeling complex
7SK snRNP	7SK small nuclear ribonucleoprotein
AP	affinity purification
MS	mass spectrometry
IP	immunoprecipitation
Y2H	yeast 2 hybrid
