# Supplementary material for: NANOG Proximity Proteomics Maps Neighborhood Hubs Linked to Mesenchymal Stem Cell Stemness and Chromatin Control
Source: Biomolecules. 2026 Apr 2;16(4):531. doi: 10.3390/biom16040531 (PMC13113505; doi:10.3390/biom16040531)
Supplement: Supplementary file 1 [file biomolecules-16-00531-s001.zip › biomolecules-4136480-supplementary-r3/biomolecules-4136480 Supplementary_Figures_r2.pdf]

Supplementary Figures

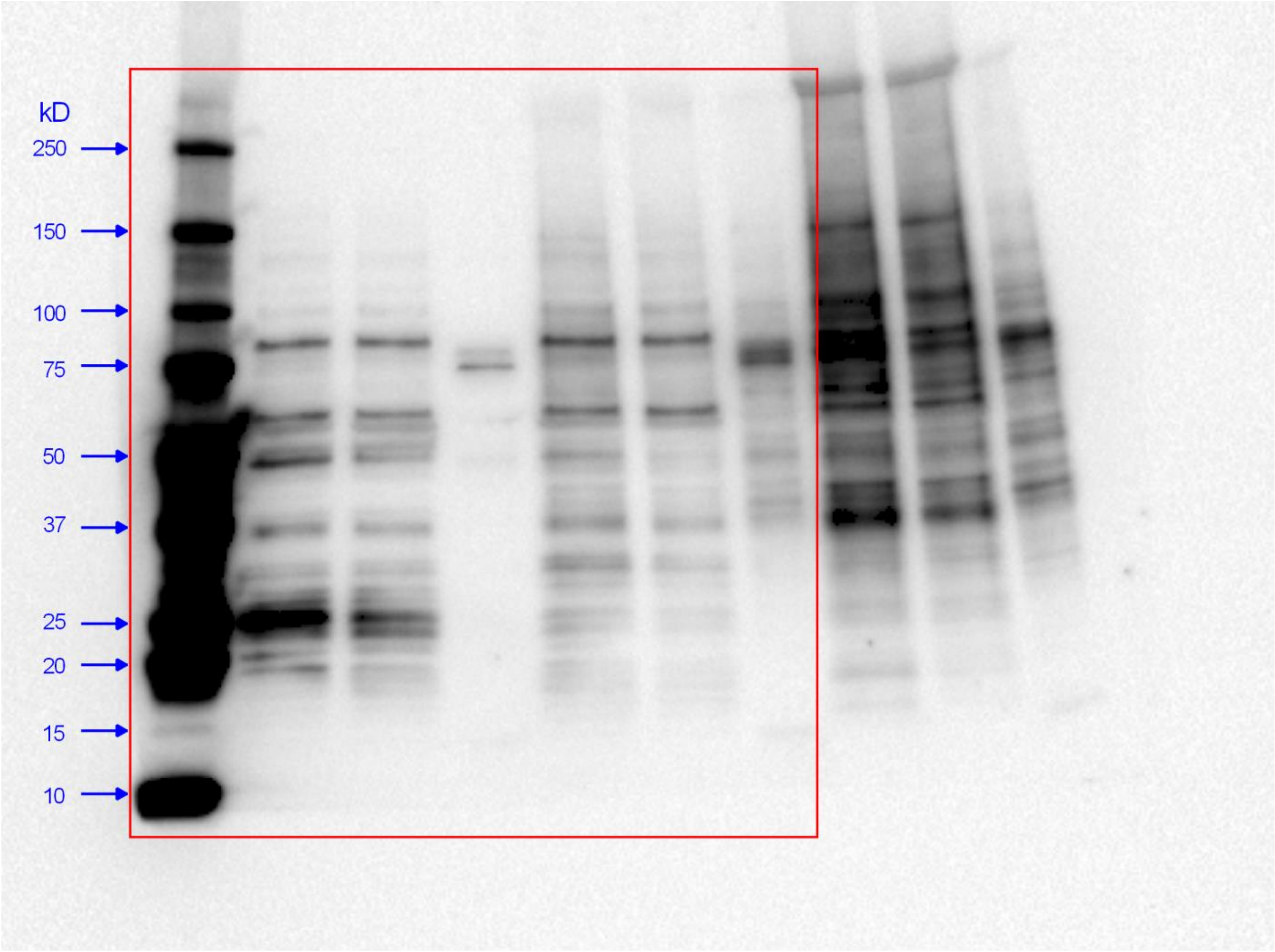

Figure S1. Original streptavidin-horseradish peroxidase (HRP) blot image corresponding to Figure 3A. The blot was detected by chemiluminescence using a ChemiDoc MP Imaging System (Bio-Rad). The highlighted region (red box) shows the Precision Plus Protein All Blue Prestained Protein Standards (Bio-Rad, Cat. #1610373), followed by total lysate, unbound fraction and streptavidin bead-bound fraction from the negative control (NC) condition, and total lysate, unbound fraction, and streptavidin bead-bound fraction from the wild-type (WT) condition.

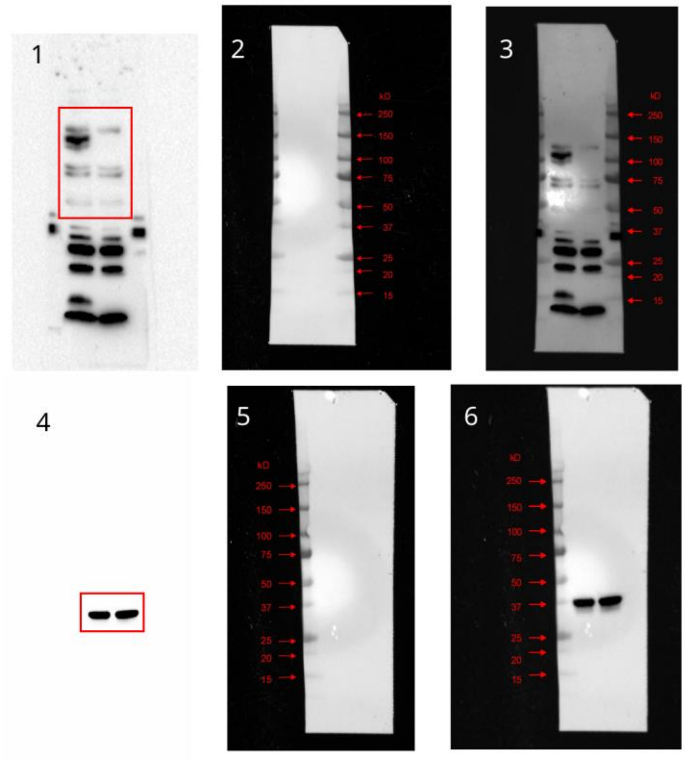

Figure S2. Original western blot images corresponding to Figure 3B. Panels 1 and 2 were obtained from the same PVDF membrane: Panel 1 shows chemiluminescent detection of NANOG, and Panel 2 shows the corresponding colorimetric image acquired to visualize the Precision Plus Protein All Blue Prestained Protein Standards. Panel 3 is a merged image of Panels 1 and 2. Panels 4 and 5 were obtained from the same PVDF membrane: Panel 4 shows chemiluminescent detection of GAPDH, and Panel 5 shows the corresponding colorimetric image for visualization of the prestained protein standards. Panel 6 is a merged image of Panels 4 and 5. All images were acquired using a ChemiDoc MP Imaging System (Bio-Rad). The samples shown correspond to mesenchymal stem cells (MSCs) expressing mApple-APEX-NANOG and untransduced MSCs. The highlighted regions (red boxes) represent the area displayed in Figure 3B.
